# Dynamic models to predict health outcomes: current status and methodological challenges

**DOI:** 10.1186/s41512-018-0045-2

**Published:** 2018-12-18

**Authors:** David A. Jenkins, Matthew Sperrin, Glen P. Martin, Niels Peek

**Affiliations:** 1 Health e-Research Centre, Farr Institute, Faculty of Biology, Medicine and Health, Manchester Academic Health Science Centre, University of Manchester, Manchester, UK; 2 NIHR Greater Manchester Patient Safety Translational Research Centre, The University of Manchester, Manchester, UK; 3 Faculty of Biology, Medicine and Health, University of Manchester, City Labs 1.0, Nelson Street, Manchester, M13 9NQ UK

**Keywords:** Prediction models, Calibration, Dynamic models, Validation

## Abstract

**Background:**

Disease populations, clinical practice, and healthcare systems are constantly evolving. This can result in clinical prediction models quickly becoming outdated and less accurate over time. A potential solution is to develop ‘dynamic’ prediction models capable of retaining accuracy by evolving over time in response to observed changes. Our aim was to review the literature in this area to understand the current state-of-the-art in dynamic prediction modelling and identify unresolved methodological challenges.

**Methods:**

MEDLINE, Embase and Web of Science were searched for papers which used or developed dynamic clinical prediction models. Information was extracted on methods for model updating, choice of update windows and decay factors and validation of models. We also extracted reported limitations of methods and recommendations for future research.

**Results:**

We identified eleven papers that discussed seven dynamic clinical prediction modelling methods which split into three categories. The first category uses frequentist methods to update models in discrete steps, the second uses Bayesian methods for continuous updating and the third, based on varying coefficients, explicitly describes the relationship between predictors and outcome variable as a function of calendar time. These methods have been applied to a limited number of healthcare problems, and few empirical comparisons between them have been made.

**Conclusion:**

Dynamic prediction models are not well established but they overcome one of the major issues with static clinical prediction models, calibration drift. However, there are challenges in choosing decay factors and in dealing with sudden changes. The validation of dynamic prediction models is still largely unexplored terrain.

## Introduction

Healthcare systems have limited resources and their budgets are being reduced [1], while there are increasing numbers of people living with one or more long-term conditions [2, 3]. This can have a negative effect on health outcomes [4], and systems therefore need to be more efficient. One way to improve efficiency is by implementing preventative measures which delay or prevent the onset of disease and increase the overall health of the population. Increased data collection in healthcare systems and availability of large-scale data sources provide an opportunity to effectively target care and resources in a data-driven way. This could also be used to guide health policies, assist in healthcare auditing and select appropriate therapies in individual patient management, along with other uses [5] to improve the healthcare system as a whole.

Clinical prediction models (CPMs) are used for diagnosis or prediction of future outcomes for individuals [6, 7] and thus have the potential to be used for decision-making and effective targeting of resources. CPMs use information about an individual at a given time, to compute the risk/probability of a future outcome; they have been increasingly used over the past two decades. CPMs are currently used to support various decisions. For example, QRISK [8] computes an individual’s risk of developing cardiovascular disease over the next 10 years, and if the individual’s risk is above 10%, then they would be considered for statins.

Over time, population demographics, prevalence of disease, clinical practice, and the healthcare system as a whole may change, meaning that predictions based on static data can become outdated and hence no longer accurate. This is known as calibration drift [9] and is one of the major pitfalls in using CPMs in practice [10]. It can lead to over or under-prescribing of treatment and, if the model is used for audit purposes, it can provide misleading results because it does not correctly adjust for case mix. QRISK [11] is updated yearly for this reason. However, this provides periodic updates, and although this is a step in the right direction, it is problematic because patients’ calculated risk changes abruptly when updates are applied, while patients’ actual outcomes do not.

It would be advantageous if models could be produced that would continuously update over time as more information is collected and made available, thus providing accurate risk predictions that respond rapidly to new information. This could reduce the use of outdated models and avoid multiple models being produced and used, reducing both time and effort. This approach is known as dynamic prediction modelling. We define dynamic models (DMs) as those which acknowledge the real time of each point, are designed to evolve over time and address the problem of calibration drift. The model could, in principle, change after a single new observation, which could be a structural change or a coefficient change. Models can evolve over time, and an individual’s risk can also change over time. Here, we focus on models evolving over time as opposed to the alternative where we observe repeated measures for an individual and observe time-varying coefficients.

Our aim was to review methods for developing and validating dynamic prediction models, in order to understand the current state-of-the-art in this field and identify unresolved methodological challenges.

## Methods

### Search strategy

The literature search was conducted in three electronic databases, MEDLINE, Embase and Web of Science. OVID was used to search the former two databases, and searches were restricted to the English language because of limited translation resources but were not restricted by publication year. The MEDLINE search terms comprise terms the authors considered to best describe dynamic prediction modelling (Table 1). The search was tailored to each database and supplemented with relevant papers that were identified from the reference list of the included papers. Further snowballing, using Google Scholar, was also performed by conducting a citation search which identified papers referencing our initial relevant paper list.

**Table 1:** Ovid search terms

1	dynamic model*.mp. [mp = ti, ab, hw, tn, ot, dm, mf, dv, kw, fx, nm, kf, px, rx, ui, sy]
2	dynamic prediction*.mp. [mp = ti, ab, hw, tn, ot, dm, mf, dv, kw, fx, nm, kf, px, rx, ui, sy]
3	clinical prediction model*.mp. [mp = ti, ab, hw, tn, ot, dm, mf, dv, kw, fx, nm, kf, px, rx, ui, sy]
4	dynamic model* prediction.mp. [mp = ti, ab, hw, tn, ot, dm, mf, dv, kw, fx, nm, kf, px, rx, ui, sy]
5	dynamic regression.mp. [mp = ti, ab, hw, tn, ot, dm, mf, dv, kw, fx, nm, kf, px, rx, ui, sy]
6	dynamic logistic regression.mp. [mp = ti, ab, hw, tn, ot, dm, mf, dv, kw, fx, nm, kf, px, rx, ui, sy]
7	model updating.mp. [mp = ti, ab, hw, tn, ot, dm, mf, dv, kw, fx, nm, kf, px, rx, ui, sy]
8	clinical prediction.mp. [mp = ti, ab, hw, tn, ot, dm, mf, dv, kw, fx, nm, kf, px, rx, ui, sy]
9	(dynamic model* and updat*).af.
10	dynamic prediction model*.af.
11	model revision.mp. [mp = ti, ab, hw, tn, ot, dm, mf, dv, kw, fx, nm, kf, px, rx, ui, sy]
12	model recalibration.mp. [mp = ti, ab, hw, tn, ot, dm, mf, dv, kw, fx, nm, kf, px, rx, ui, sy]
13	1 or 2 or 4 or 5 or 6 or 9 or 10
14	3 or 8
15	13 and 14
16	7 or 11 or 12
17	15 or 16
18	dynamic.mp. [mp = ti, ab, hw, tn, ot, dm, mf, dv, kw, fx, nm, kf, px, rx, an, ui, sy]
19	14 and 18
20	17 or 19

### Selection of studies

A two-stage screening process was conducted by one author (DJ) to assess the relevance of studies and was applied after the initial search and again after the two snowballing approaches. The first stage consisted of screening the titles and abstracts of citations to exclude articles that did not meet the inclusion criteria. The eligible criteria for inclusion were original methodological peer-reviewed journal articles which considered (1) dynamic prediction models (DPMs), (2) model updating methods that could be performed in real time, or (3) model coefficients as functions of time. Exclusion criteria were determined in advance and included conference proceedings, papers with methods that could not change over time or update in real time, static prediction models and models that only consider a single time point (e.g. models for cross-sectional data). Dynamic survival models were also excluded because they do not fall under our definition of dynamic prediction. Applied research, without any methodological work, was excluded because our interest was around the current state-of-the-art and methodology in the area.

### Extraction

We evaluated papers on two general domains: modelling methods and validation and evaluation. We extracted each method we considered to meet, or have the potential to meet, our definition of dynamic modelling. For validation, we extracted how the models implemented were evaluated. For all the methods found during the search, we also extracted any modelling challenges and further work discussed by the authors and provide our suggestions for the future work needed in the area.

No specific study measures or synthesis were calculated across studies.

## Results

**Fig. 1:**
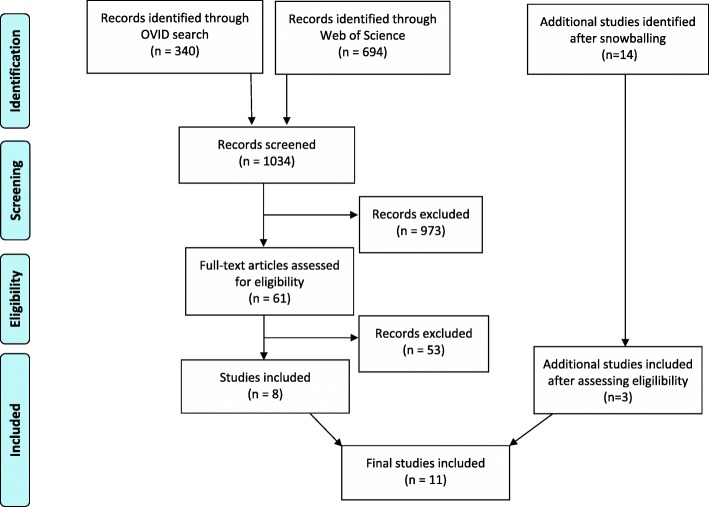
PRISMA flow diagram of included studies

Our initial search resulted in 1034 papers, with 61 considered potentially relevant after abstract and title screening. After full article screening, 8 were identified for which information was extracted and snowballing was taken place. An additional 14 papers were then considered relevant, but after screening, only 3 were included for which information was extracted. Hence, in total, 11 papers were deemed relevant for final inclusion (see Fig. 1).

Seven methods were reported across 11 papers which could be used to deal with calibration drift in prediction models (see Table 2). These can be split into three categories: discrete model updating, Bayesian model updating and varying coefficient modelling.

**Table 2:** Tick table of methods included in each paper

Author	Modelling methods						
	Discrete model updating				Bayesian model updating		Varying coefficient modelling
	Intercept update	Overall slope update	Individual slopes update	Model revision	Bayesian dynamic modelling	Bayesian model averaging	
Fan							✓
Finkelman					✓		
Hickey				✓	✓		
Hoover							✓
Janssen	✓	✓	✓	✓			
McCormick					✓	✓	
Raftery					✓	✓	
Siregar	✓	✓		✓	✓		
Steyerberg	✓	✓	✓	✓			
Su	✓	✓	✓	✓	✓	✓	
Van Houwelingen			✓				

To illustrate the approaches, we will focus on prognosis and consider a regression model with either a continuous or binary endpoint at a fixed point in time. A response *y*_*t*_ is observed for an individual at a time *t* = (*t*_1_, …, *t*_*n*_), and a vector of predictors *x*_*t*_ = (*x*_*tk*_ : *k* = 1, …, *K*) such that:

1 $$ g\left(E\left({y}_t\right)\right)={\beta}_0(t)+{\beta}_K(t){x}_t, $$

for link *g*, where *β*_0_(*t*) is the intercept and *β*_*K*_(*t*) is a vector of the regression coefficients for the *K* predictors at time *t*.

Equation 1 is a general form of a dynamic prediction model, but the methods described in the literature vary on how to estimate the coefficient functions *β*_0_(*t*) and *β*_*K*_(*t*) and update the model. Below, we outline each of the methods found in the literature, followed by a discussion of the various challenges highlighted within the papers.

### Modelling methods

#### Discrete model updating—model recalibration and revision

Discrete model updating methods use new data over time to update the model. Using a single observation or small group of observations can result in an unstable and less accurate model. Hence, these methods are updated in batches at set times, for example, each month or year, to ensure a sufficient amount of data is collected and used for the update. We denote these batch times as *T*_*j*_ = (*T*_1_, …, *T*_*B*_) where *B* ≪ *n*.

Four discrete updating methods are discussed in the final included papers; ‘Intercept update’, ‘overall slope update’, ‘individual slopes update’ and ‘model revision’. All four methods consider a frequentist approach.

The ‘intercept update’ method [12–15] fits a regression model to the new data, at updating batch time *T*_*j*_, using the linear predictor of Eq. 1 as an offset. This recalculates a new intercept, *β*_0_(*T*_*j*_) with *β*_*K*_(*T*_*j*_) = *β*_*K*_ remaining constant over time.

‘Overall slope update’ [12–15] re-estimates both the intercept and an overall slope *α*(*T*_*j*_) for each update time *T*_*j*_. This factor is used to proportionally adjust the original coefficients and thus creates a new predictor-outcome association *β*_*K*_(*T*_*j*_) = *α*(*T*_*j*_)*β*_*K*_(*T*_*j* − 1_) and a new intercept, *β*_0_(*T*_*j*_).

‘Individual slopes update’ [13–16] is a two-step method where the overall slope updating is first used and then a subset of the coefficients, which are statistically different in the new data compared with the historic data, are re-estimated. Thus, *β*_*K*_(*T*_*j*_) = *α*(*T*_*j*_)*β*_*K*_(*T*_*j* − 1_) + *γ*_*K*_(*T*_*j*_) where *γ*_*K*_(*T*_*j*_) is a vector of length *k* which has zeros located in the elements corresponding to the parameter estimates that are not re-estimated. The choice of which variable coefficients to re-estimate can be decided by a likelihood ratio test, stepwise variable selection or obtaining expert opinion. A special case [12–17] would be to update all model coefficients and not only those that are statistically different. Hence, all prognostic effects are updated and the original CPM is only used to select the covariates included in the updated model. After revision, shrinkage can be conducted, where the coefficient estimates are shrunk towards the recalibration estimates [13–15]. This can be done, for example, using ridge regression [18, 19].

‘Model revision’ [13, 15] considers adding predictors into the model. This method re-estimates existing coefficients as in the above approaches, but also tests if any additional predictors now have a statistically significant effect in model fit by performing likelihood ratio tests in a forward stepwise variable selection manner. This builds a new model.

2 $$ g\left(E\left({y}_t\right)\right)={\beta}_0(T)+{\beta}_L(T)\ {x}_t $$

Where *L* is the total number of predictors in the updated model at the time *T*_*j*_, such that, *L* ≥ *K* and *L* − *K* is the number of additional predictors added to the model at time *T*_*j*_. This model is applied for individuals *t* such that *T*_*j*_ ≤ *t* < *T*_*j* + 1_.

#### Continuous model updating—Bayesian updating

Two continuous updating methods are discussed in five of the final included papers [12, 13, 17, 20–22]. The first method is known as Bayesian dynamic modelling, and the second, known as dynamic model averaging, is a generalisation of the first. In both methods, the information obtained from past data is used as prior information and combined with the new data to obtain updated estimates. Thus, the updating (posterior) equation is proportional to the product of the likelihood (at time *t*) and the prior (prediction equation at time *t*−1),

3 $$ p\left(\ {\beta}_K(t)\ |\ {Y}_t\right)\propto p\left(\ {\beta}_K(t)\ |\ {Y}_{t-1}\right)p\left(\ {y}_t|\ {\beta}_K(t)\ \right)\propto \mathrm{Prior}\ \mathrm{x}\ \mathrm{Likelihood} $$

where the prediction equation (prior) is obtained through Kalman filtering by supposing $p\left(\upbeta \left(t-1\right)|\ {Y}_{t-1}\right)\sim N\left(\widehat{\upbeta}\left(t-1\right),{\varphi}_{t-1}\right)$, where *Y*_*t* − 1_ = {*y*_1_, …, *y*_*t* − 1_}. This results in the prediction equation.

4 $$ p\left({\beta}_K(t)\ |{Y}_{t-1}\right)\sim N\left({\widehat{\upbeta}}_K\left(t-1\right),{R}_t\right);{R}_t={\varphi}_{t-1}+{W}_t,. $$

where *W*_*t*_ represents the covariance matrix.

We can also introduce a forgetting factor, *λ*_*t*_, such that $$ {R}_t=\raisebox{1ex}{${\varphi}_{t-1}$}\!\left/ \!\raisebox{-1ex}{${\lambda}_t$}\right. $$. This down weights (or decays) historical observations so they have less influence/weight than new data by essentially inflating the variance of the prior. Typically, *λ*_*t*_ is constant over time, and close to 1. In principle, the forgetting factor could change over time, but this has yet to be done in practice. A forgetting factor of 0.99 was used in one study [21], while another [13] performed sensitivity analysis using different values for the forgetting factor. Su et al. [13] suggests that *λ*_*t*_ can be selected using an auto-tuning procedure at each time point which could result in a time-varying forgetting factor. However, this would result in a much higher computation load.

Two advantages are discussed in using the forgetting factor. The first is that the model becomes less computationally demanding than when forgetting is not applied, which can make the dynamic model more feasible to use in practice. The second is that the model relies less on the historical data. If the model coefficients are changing over time, then giving a lot of weight to past data may decrease prediction accuracy. Also, the historical data used for the prior could anchor the results and provide inaccurate predictions.

The first method described is for a single model case, but if there exist multiple models *M*_1_, …, *M*_*m*_ implemented at the same times, then the above approach can be applied simultaneously to each model. We can then combine each of the *m* models together to create one final model, thus resulting in dynamic model averaging (DMA). In DMA, a weighted average of models is used at each time point, where the ‘better’ models contribute more weight in the final model and the weights can vary over time. One major advantage of this approach is that it allows parameters to be down-weighted/excluded and emerging factors to become present over time. Hence, there is extra flexibility in this approach that the others do not have and forgetting can also be applied within DMA.

All of the above methods, both continuous and discrete, are two-step approaches in which the initial CPM is computed using the first batch of data and that model is subsequently updated in light of new data. The initial model will generally fix which parameters are included within the model, although, as described above, there is a discussion in the literature [23] about adding or deleting predictors during the updating. The majority of studies set a specific time interval where all the data within that window would be used for the next update. One study [21] had data to perform monthly updates but another [12] only considered updating yearly, and one [10] considered updating models on either a monthly, yearly or 2 yearly basis. Finkelman et al. [22] was the only paper to consider the batch as observation numbers and not the length of time. They considered 250, 500, 1000 and 5000 for the updating batch numbers and concluded the results were ‘fairly insensitive to changes’ in the size of the update. Some papers [10, 22] have suggested, for the discrete methods, that a sufficient number of new data is needed in each batch to ensure enough is obtained for accurate and stable predictions. Step one of these models will not always consider the same time period as step two (model updating). For example, one of the models, Hickey et al. [17], conducts, uses a first step of 12 months to create the initial CPM but then uses monthly updates for step two.

Some of the studies also compared which of the methods performed best. However, not all methods were included in each paper. Raftery [20] used mean square error (MSE) and maximum absolute error (MAE) to compare the Bayesian models. After 200 sample updates, the models become stable and differences between models become smaller than in the initial sample updates where the DMA performs better because ‘it’s more adaptive’. Finkelman also used MAE to compare models, but to improve interpretability, the ‘relative improvement’was computed, which is the improvement of the current model compared with the intercept-only model. McCormick, on the other hand, used the Brier score to compare model performance.

#### Varying coefficient model

Varying coefficient models [24] (also known as functional response models) were developed to explore dynamic patterns in data. These are particularly useful when we encounter multiple data from the same individuals over time, known as longitudinal data, and/or have data changing over time, known as functional data. Varying coefficient models are often used to model longitudinal data, for example, the risk of HIV after birth [25] and *β*_*K*_(*t*) is modelled as a function of time from birth. We can also use it as an approach to dynamic modelling in which the relationship between predictors and the outcome variable is described as a function of calendar time. This approach has been used in other areas, such as economics, but not yet in healthcare.

Following the form of Eq. 1, in this case, we have *β*_0_(*t*) and *β*_*K*_(*t*) = (*β*_1_(*t*), …, *β*_*K*_(*t*)) as functions of time which are assumed to be smooth. Hoover et al. [25] presents three ways the coefficients can be estimated: kernel, polynomial and smoothing splines.

A special case of this method is where only the intercept is dependent on time. Eq. 1 would then become

$$ g\left(E\left({y}_t\right)\right)={\beta}_0+{\beta}_K{x}_t+{\beta}_{time}t, $$

where the betas are no longer functions of time and *β*_*time*_ adjusts the intercept for observed calibration drift in the development dataset, i.e. *β*_0_(*t*) = *β*_0_ + *β*_*time*_*t* . This is arguably the simplest approach to overcome the problem of calibration drift, but to the best of our knowledge, it has not been applied in healthcare settings to improve calibration. Compared with the previous methods, the varying coefficient model does not regularly update at each time but rather attempts to estimate the complete function of the coefficients over time given data up to a certain time point. Hence, this method does not view data as a stream but rather assumes all data are available over time and then estimates *β*_*K*_(*t*). No study considering varying coefficient models also considered discrete or continuous updating approaches. A comparison between the different methods has yet to be explored.

All included papers discuss dynamic models as a way of using all the data available to create models that are evolving over time and have the flexibility of adapting to a changing landscape over time. The discrete and continuous updating models use current/new data to update past knowledge, rather than using a static time frame and assuming the prediction model remains the same over time. However, the weight applied to the historic data varies. On the one hand, all data, historic and new, is used equally. On the other hand, the historic data may be given no weight in the update, so only the new data is used to update the model. These are just two extremes, and dynamic model updating can be anywhere within this space. The functional varying coefficient models differ because they are not updating over time. These models use the complete data available to estimate the coefficient function over time in order to provide future predictions. However, they have the potential to be updated using discrete updating, but this has yet to be explored.

### Model validation

Once a model has been computed and selection of appropriate predictors has taken place, it is not sufficient to assume the model is accurate and predicts well. We therefore need to formally validate our models. For static CPMs, cross-validation and bootstrap validation are the recommended methods over split sample or external sample validation [12], but validation is more complex when it comes to DPMs. The literature around validating a dynamic CPM is much less established, meaning that it was not possible to identify different validation techniques for each of the dynamic modelling methods separately.

Siregar et al. [12] and Su et al. [13] assess calibration and discrimination in all of their models. Both validate their models in subsequent years (after model updating has stopped), but in reality, the model would continue updating and so a way is needed to validate in this framework to provide real-time validation without using the same data that is then used for the model. Su et al. [13] also note that because validation is conducted at a separate time to the model, then their validation constitutes transportability rather than validation. Split sampling could be performed, where part of the sample at each update is used to validate the updated model, but this was not explored in any of the papers and would need doing in a dynamic way which could add to the computational aspect of the models. Van Houweingen [16] conducts a split sample validation on the original CPM and uses this to determine if an update is needed as the new data is collected; however, validation of the updating model was not undertaken. There would also be a lag time from determining if a model is valid, such that, the model would possibly have been updated many more times. McCormick [21], on the other hand, designed their model for a setting when data is not stored and so validating can be an issue here. They suggest maximising the average one-step-ahead prediction by updating a tuning parameter, but not through numerical optimisation because of computational infeasibility. Using an Occams window [26, 27] approach was also discussed as a possible solution to computational problems. This would consider a smaller model space at each time (after time 1) by using a subset of all possible models based on a cutoff value for each models weight contribution at the previous time. However, none of these methods have been formally implemented in any of the included papers or across healthcare, although this has been applied within economics [28].

Hickey et al. [10] produced time series plots of the beta values to obtain inferences of the association between the outcome and risk factor. This allowed for comparison of methods, as well as the ability to visually detect any abrupt changes. Although this provides a better understanding of how the models are working, it is not a formal way in which to validate, test or compare models. Hickey et al. [10] acknowledge that not performing validation was a limitation of the study and suggests that to do so, one would need to compute and monitor the model’s discrimination over time, in a continuous way. Conducting time series on the coefficients could potentially be used to detect patterns in the coefficient estimate over time or even be used as a way to predict future beta estimates which could then be compared to the DM predictions, but either has yet to be explored.

Therefore, validation is a clear issue in this area and was only used in a small number of studies which mainly considered the discrete approaches.

### Other challenges

All of the methods described above assume a steady change in the model coefficients over time. However, sudden large changes are possible and could result in poor model performance. These changes can occur for many reasons, such as a change in policy, introduction of new interventions, a change in data collection, or the introduction of clinical decision support that is based on the CPM. An example of a step change in clinical practice is the introduction of less invasive coronary surgery [10]. This change in surgery, along with a change in the case-mix of the population undergoing cardiac surgery, resulted in the EUROSCORE CPM [29] largely over-predicting patient risk [7]. One way to model these changes in a CPM would be to include a time factor but it has yet to be discussed in the literature how well dynamic models react to these changes and which models provide the most accurate predictions and should be used in these circumstances. However, this assumes that a step change is anticipated for a known reason. However, in practice, it is not always anticipated or known. Therefore, it would also be advantageous to account for, and model, unexpected step changes. McCormick et al. [21] suggest that when these occur, a smaller forgetting factor should be chosen to allow for these changes. However, the windowed approach in Hickey et al. [17] is used to dampen any abrupt changes. Step changes have the potential to impact model accuracy and being able to identify them, as well as knowing how to deal with them, could have great benefit. There is currently little work discussing what to do when they occur and how to detect or define a true step change. Analysing the impact of these changes (with various magnitudes and frequency) on model performance and understanding how best to weight past data (if at all) when they occur would be largely beneficial for future work. Also, the ability to detect step changes would be valuable and could be used to either identify when models need to be updated or inform the user a change has occurred and investigation into the data is needed.

Finally, computational complexity was discussed as a limitation of DMs, but only two papers [20, 22] formally considered computation time. Finkelman et al. [22] discuss that the computation time linearly increased with the number of updates, but around the same number of subjects were included in each update and computation time could vary if the numbers varied across iterations. Raftery et al. [20] discuss that although DMs and DMA do increase the computation time, they are still well within a range for practical application. In a large system when updating is to be applied when each new data point is collected, then this could be problematic if the computation time associated with updates exceeds the time between subsequent data points. Continuous model updating is then not feasible.

The software is available to perform dynamic modelling; the dma [30] and fda [31] packages in R can be used for continuous updating and the varying coefficient methods, respectively. To our knowledge, no package is available for discrete updating, but it can easily be programmed manually in many software packages. Extension of these, along with user-friendly tutorials, would aid widespread implementation into the clinical setting.

## Discussion

In this study, we conducted a literature review which has identified three main types of dynamic modelling, with the main differences between the methods emerging in relation to how the coefficients are estimated. Our review has enabled us to draw together all the methods within one paper and highlight gaps in the literature for future research. Discrete and continuous updating have been used a small number of times within the healthcare setting to address the issue of calibration drift. These methods update the model over time, which provides the dynamic aspect of these models. We have also identified an additional method, varying coefficient modelling, that could be used in healthcare but has yet to be implemented for dynamic prediction in this setting. This method differs in comparison to others as it does not update but uses the data up to time *t* to estimate the function for each coefficient in the model over time. The continuous updating and varying coefficient methods both assume a smooth function over time and discrete updating differs by assuming discrete changes. These dynamic prediction models have the potential to be extremely useful but currently have limited exposure to healthcare problems, and validation of these models in practice is challenging. Further work is needed to develop ways to validate these models and assess how these models perform under different healthcare settings and scenarios.

To our knowledge, only two other studies have performed a review of dynamic modelling methods. Su et al. [13] describes both the discrete and continuous updating methods and then applies them to a clinical data set, updating on a monthly basis. Comparisons of model performance and accuracy of future predictions were then made. Siregar et al. [12] also describe the discrete and continuous updating methods, excluding dynamic model averaging. The methods were then applied to a cardiac data set by updating the EUROSCORE model and comparing model discrimination across all methods. Overall, our work is consistent with these two papers but extends the findings by conducting an up-to-date literature search and includes the use of varying coefficient modelling as a possible method to maintain model performance over time. Comparisons of the intercept updating method with different updating times and population size were compared with the standard continuous updating method by Hickey et al. [17]. This work compares the methods in a real-world situation and discusses the limitations of the methods, but it is not a complete review of dynamic modelling. Our review draws together all methods in the literature and identifies gaps in the literature but does not provide practical examples and direct comparisons of all the methods found.

The most pressing problem to address, which we have highlighted in this study, is that of validation. The purpose of any model validation is collected incremental evidence that the model works satisfactorily in populations where it is applied—thus provided trust among its potential users and enabling adoption [32, 33]. Many well-established (static) prognostic models, such as the Apache IV model [34] for predicting mortality in critically ill patients, were validated in numerous studies before they were broadly adopted in clinical practice. Because dynamic prediction models are moving targets, it is fundamentally impossible to follow the same approach. We can validate each of the individual iterations, but by the time that users have taken notice of the validation results, the model will have already progressed to a next iteration and those results might be outdated. So, to enable a similar mechanism that instills trust and fosters adoption, validation methods are needed that can provide evidence of good performance of the entire dynamic ‘system’. These methods should convince us that both the initial model and all its future iterations have good performance, regardless of the new data points that are used for updating.

Future work would also benefit from assessing the impact of step changes, as well as the impact size and frequency of updates could have on predictions. A close test procedure has previously been used [35] to select which discrete updating method should be used when updating your model. However, this has only been used for transportability to a new population, opposed to updating regularly over time. Exploring this method to address calibration drift, as well as, extending the method to include Bayesian updating and decide when/if updating should occur would be extremely useful and increase the utility of the approach. Testing and comparing these dynamic models in more complex data structures, such as clustered data, would also be beneficial. This could be done with the use of random effects or generalised estimating equations, as previously suggested [22, 25]. Also, only a small number of studies have applied and considered these dynamic modelling methods for use within healthcare, with the majority of applications only considering the discrete updating methods [36] and focusing on transportability for models to different populations [37, 38] rather than using the methods discussed to address temporal changes over time. Therefore, more practical examples and comparisons of the methods found are warranted for further work. This would help aid the broader adoption of these methods into clinical practice, which is a current issue with CPMs as a whole. While this is not confined to dynamic prediction models, this is a common problem with prediction models and refinements, such as improved reporting and better use of existing CPMs (e.g. a focus on external validation rather than de novo development) could improve the adoption of CPMs in clinical practice. Also, incorporating models into hand-held technology (e.g. mobile apps to allow calculation of complex models a patient’s bedside) and extending the methods into software with user-friendly tutorials would be of value.

Because dynamic prediction models are an emerging field and not a well-established concept, different authors may have used different terminologies to describe dynamic prediction models; further, there are currently no MeSH terms for these methods and this could have resulted in some studies not being captured within our search. Our search focussed on the methodological papers, and it was not possible to go through all of the applied work. This may have resulted in some methods, or adaptations of existing methods, not being captured within our search. Nevertheless, we believe that we have identified the main methodological approaches to dynamic model development, updating and validation.

Although the focus of this review was in methods accounting for temporal differences over time, some of the methods and issues raised would apply to geographic or contextual updating, for example, where a model is to be used in a different population to which it was originally developed. Also, although we restrict our attention to prognostic models, the findings are generalizable to diagnostic modelling.

## Conclusion

Several statistical methods for creating dynamic prediction models have been described in the literature. These methods are well developed but their application to real-world clinical prediction problems is sparse and no dynamic prediction models have been deployed in clinical practice. Validation of dynamic prediction models is an unresolved issue that needs to be addressed urgently.

## Abbreviations

CPM: Clinical prediction model
DM: Dynamic model
DMA: Dynamic model averaging
